# Emotion dysregulation and psychopathological symptoms in non-clinical adolescents: The mediating role of boredom and social media use

**DOI:** 10.1186/s13034-023-00700-0

**Published:** 2024-01-06

**Authors:** Sara Iannattone, Selene Mezzalira, Gioia Bottesi, Michela Gatta, Marina Miscioscia

**Affiliations:** 1 Department of General Psychology, University of Padua, via Venezia 8, Padua, 35131 Italy; 2 Department of Engineering, University of Basilicata, Potenza, Italy; 3 Department of Women’s and Children’s Health, University of Padua, Padua, Italy; 4 Department of Developmental Psychology and Socialization, University of Padua, Padua, Italy

**Keywords:** Adolescents, Boredom, DERS, Emotion regulation, Internalizing and externalizing problems, Social media use

## Abstract

**Background:**

Emotion dysregulation, boredom, and problematic social media use are well-known vulnerability factors for psychopathology during adolescence; nevertheless, the interplay between these factors remains underinvestigated in the literature. Therefore, the present cross-sectional study aimed to explore the mediating role of boredom and problematic social media use in the relations between emotion dysregulation and both internalizing and externalizing problems in a non-clinical group of Italian adolescents.

**Method:**

721 students (64.6% girls; *M*_age_ = 15.49 years ± 1.82) were involved and completed self-report tools assessing emotion dysregulation, boredom, problematic social media use, and psychopathological symptoms. Path analysis was used to test whether boredom and problematic social media use mediated the relation between emotion dysregulation and psychopathology, distinguishing between internalizing and externalizing problems.

**Results:**

Path models showed that emotion dysregulation predicted both internalizing and externalizing problems, as well as boredom and problematic social media use. Importantly, boredom mediated the associations between emotion dysregulation and both psychopathological dimensions, while problematic social media use mediated only the relation with externalizing problems.

**Conclusions:**

Our results highlight that the influence of emotion dysregulation on psychopathology can manifest through different paths, leading to specific symptomatology based on interactions between various variables. In particular, boredom seems to be a transdiagnostic factor for psychopathology in adolescence, whereas problematic social media use would be a dimension-specific factor. The practical implications of these findings are discussed.

## Introduction

Adolescence is characterized by profound changes primarily attributed to puberty onset, which encompasses physical maturation, cerebral transformations, and general psychosocial shifts [26]. This developmental metamorphosis involves an increased reliance on cognitive control capabilities, specifically deploying top-down effortful control to regulate attention, emotion, and behavior, thus aligning with the cognitive goals typical of adult functioning [10]. The trajectory of pubertal maturation leads adolescents to attain greater autonomy from their parents [68], while friendships evolve towards greater intimacy, supportiveness, and communicativeness [21]. Adolescence is also a time of self-exploration [31], especially during late adolescence, when individuals begin to develop more abstract, differentiated, and organized self-concepts compared to childhood [68]. Importantly, all these changes make adolescence a stage marked by significant psychopathological vulnerability [66]; indeed, both internalizing and externalizing problems are particularly frequent during this developmental phase. Symptoms of the internalizing dimension include anxiety, depression, withdrawal, and somatization, while symptoms of the externalizing dimension encompass aggression, opposition, substance use, hyperactivity, and rule-breaking behavior [1, 2, 76]. In light of this, it appears paramount, for both preventive and treatment purposes, to delve deeper into the correlates of internalizing and externalizing problems in this age group [6, 17, 50].

One of the main constructs studied in relation to psychopathology is emotion regulation, which is characterized by a developmental nature. Indeed, emotion regulation strategies undergo a transformative evolution, progressing from dyadic co-regulation – mainly used by infants to self-regulate through to the presence of a caregiver – to more autonomous ways of self-regulating [28]. Research has shown that children gradually enhance their efficiency in emotion regulation as they get older [43], also thanks to the maturation of the prefrontal cortex, a pivotal structure for emotional control [57]. Moreover, the path toward autonomy during adolescence is marked by significant neuronal and endocrinological changes that result in increased emotional reactivity [64]. Simultaneously, adolescents need to cultivate flexible emotional regulation mechanisms in response to the several environmental challenges they encounter [26]. However, according to the “maladaptive shift model” [86], emotion regulation abilities would undergo a dysfunctional change in adolescence, since increased emotional reactivity and instability may deplete cognitive control, resulting in a reduction in the use of adaptive emotion regulation strategies and an increase in the reliance on maladaptive ones [25].

Gratz and Roemer [40] conceptualized emotion regulation as a multidimensional construct involving various dimensions such as awareness/understanding and acceptance of emotions, the capacity to control impulsive behaviors when experiencing negative emotions, and the flexible use of appropriate strategies to modulate emotional responses; in contrast, emotion dysregulation denotes an individual’s inability to control or modulate their emotional states. Emotion dysregulation represents a transdiagnostic vulnerability factor for various types of psychopathologies [4, 24, 34, 71], with particular relevance during adolescence. For example, McLaughlin et al. [54] found that difficulties in emotion regulation among adolescents predicted subsequent changes in anxiety-related symptomatology, aggressive behavior, and problematic eating habits. Additionally, a recent meta-analysis conducted by Helland et al. [45] reported that maladaptive emotion regulation strategies were implicated in both internalizing (e.g., depression and anxiety) and externalizing problems (e.g., conduct disorder, substance abuse, eating disorders, suicide, and risky behavior) among different adolescent groups.

Nevertheless, although the relation between psychopathology and emotion dysregulation in adolescence is widely supported by the literature, there is a notable gap in understanding the factors that may play a role in such a relation. In this regard, two constructs worthy of further attention could be boredom and social media use.

### Boredom, psychopathology, and emotion dysregulation

Boredom refers to an unpleasant affect characterized by the feeling of wanting something without specifically knowing what, accompanied by the inability to find sufficiently stimulating and satisfying activities [32, 78]. Boredom can be alternatively regarded as a typical feature of human nature, a dispositional trait, a reaction to monotonous environmental conditions, or a symptom of a specific psychological disorder [65]. Moreover, it has been conceptualized as a multidimensional construct encompassing factors such as lack of engagement, negative affectivity, perception of time passing slowly, and difficulties concentrating [3].

Boredom seems to be a common feeling among adolescents [3, 33, 61], with estimates suggesting that 91–98% adolescents have experienced boredom at some point in their life [22]. Moreover, the experience of boredom among adolescents has shown an increasing trend in recent years, especially among girls [82]. Crucially, boredom in adolescence can be a risk factor for several psychological problems of both the internalizing (e.g., anxiety, stress, somatization, interpersonal sensitivity, obsessive-compulsive tendencies, apathy, and anhedonia) and externalizing spectrum (e.g., delinquency, aggressiveness, substance use, risky driving, and problematic social media and smartphone use) [16, 33, 67, 69].

Pertaining to the relation between boredom and emotion dysregulation, few studies are available to date, many of which have considered a specific dimension linked to emotion dysregulation, namely alexithymia. For example, elevated levels of alexithymia were found to be related to high levels of trait boredom in a sample of undergraduate students [30]. Moreover, Liu et al. [51] found a significant and positive association between boredom propensity and alexithymia in a group of Chinese college students, with boredom propensity partially mediating the effect of alexithymia on Internet novel addiction. To our knowledge, only two works, both conducted on adult samples during the COVID-19 pandemic, have considered emotion dysregulation as conceptualized by Gratz and Roemer [40]. A longitudinal study by Bambrah et al. [11] showed that difficulties in understanding emotions and accomplishing goal-directed tasks when experiencing negative emotional states predicted greater levels of boredom at a later time point. The authors hypothesized that people who struggle to understand their feelings could also have difficulties expressing and articulating their desires - which is a crucial precondition for becoming engaged - and this could give rise to boredom. Furthermore, individuals with an impaired ability to initiate and maintain goal pursuit could be more likely to feel unengaged and, therefore, experience boredom. Finally, a cross-sectional study by Weybright et al. [81] pointed out that higher levels of emotion regulation difficulties predicted higher levels of boredom proneness.

As it can be noticed, although boredom and emotion dysregulation appear to be somehow associated, little empirical effort has been directed toward the investigation of this topic in adolescence, thus calling for further research.

### Problematic social media use, psychopathology, and emotion dysregulation

Internet use has become pervasive in the life of adolescents [52, 53], attracting heightened scientific attention and investigation into its correlates. Adolescents, in particular, are avid users of modern technologies, and their psychosocial development is significantly shaped by the engagement in Internet-related activities, which influence aspects such as self-presentation and self-disclosure [74]. Nonetheless, an excessive use of the Internet may have adverse effects on academic performance, familial connections, and emotional well-being [72]. Griffiths and colleagues [42] distinguished between generalized problematic Internet use and specific online activities such as gaming and shopping. Problematic Internet use refers to the individual’s engagement in online compulsive behaviors that, among adolescents, mainly involve social media use [37, 77, 85]. Specifically, problematic social media use has been defined as “being overly concerned about social media, driven by an uncontrollable motivation to log on to or use social media, and devoting so much time and effort to social media that it impairs other important life areas” ([8], p. 4054). Research has indicated a growing trend of problematic social media use among young people [75], potentially fueled by the platforms’ facilitation of critical socio-developmental tasks, including social interactions, ideas sharing, and identity development [48]. In addition, the social media environment has the potential to trigger the biological systems responsible for the adolescents’ increased sensitivity to social feedback and rewards [63], as well as to enable the broadcasting of various aspects of their personality, interests, and identity [73]. Crucially, however, problematic social media use is a significant risk factor for adolescent mental health; for instance, it was found to be related to low self-esteem [9], loneliness, poor sleep quality [83], and both internalizing and externalizing psychological disorders [7, 49, 56, 62, 84].

Emotion dysregulation is one of the main mechanisms potentially underlying problematic social media use and its negative consequences. For example, research has linked problematic social media use to difficulties in impulse control and goal-oriented behavior (both facets of emotion dysregulation) in young people [80]. Emotion regulation deficits have also been considered as precursors to technology-related behavioral addictions (including problematic social media use) in adolescence [37]; more specifically, according to Gioia et al. [36], problematic Internet use might function as a coping strategy to compensate for emotion regulation deficits. Moreover, it has been hypothesized that social media use can serve as a means of emotion regulation, having the function of overregulating positive affect and/or underregulating negative affect [18]. Therefore, problematic Internet and social media use may represent (maladaptive) emotion regulation strategies, contributing to significant social and psychological problems [70]; for example, maladaptive emotion regulation strategies have been identified as mediators in the association between social anxiety and both problematic social network and smartphone use in a group of non-clinical adolescents [87].

### The present study

As outlined above, emotion dysregulation, boredom, and problematic social media use represent well-established vulnerability factors for internalizing and externalizing problems in adolescents [33, 49, 69, 84]. However, the interplay between such factors remains unclear, as no prior scientific research has investigated them jointly. Based on these premises, the primary aim of the current research was to explore the role of boredom and problematic social media use as mediators in the relations between emotion dysregulation and both internalizing and externalizing problems in a non-clinical group of Italian adolescents. The importance of considering internalizing and externalizing problems separately lies in the possibility of distinguishing between dimension-specific factors and mechanisms (i.e., those that intervene only in the relation between emotion dysregulation and one of the two psychopathological dimensions) and transdiagnostic factors (i.e., those that are involved in the relation between emotion dysregulation and both dimensions). In terms of clinical practice, this can have valuable implications, as it would enable the implementation of tailored preventive and treatment interventions for adolescents.

## Hypotheses

The following hypotheses were formulated:

1. High emotion regulation difficulties were directly related to high levels of internalizing and externalizing problems [45, 54], as well as to high levels of boredom [11, 81] and problematic social media use [37, 80, 87];
2. High levels of boredom and problematic social media use predicted high levels of internalizing and externalizing problems [33, 49, 69, 84].

Finally, pertaining to the mediating role of boredom and problematic social media use in the associations between emotion dysregulation and internalizing and externalizing problems, no specific hypotheses were formulated due to the absence of previous evidence in this regard. Therefore, the current investigation aims to contribute novel insights into these complex relations.

## Methods

### Participants and procedure

The sample consisted of 721 White adolescents living in Northern and Southern Italy: 250 boys (34.7%) and 466 girls (64.6%) aged 13 to 19 years (*M* = 15.49, *SD* = 1.82). In particular, 68.1% lived in an urban area, while 31.9% in a rural area. Pertaining to school, 22.5% attended the Italian third class of a lower secondary school (5th grade), while the remaining percentage were recruited in upper secondary schools. Specifically, among the latter, 20.2% attended the Italian first class (6th grade), 22.7% the second class (7th grade), 20.9% the third class (8th grade), and 36.2% the fourth and fifth classes (9th grade). In terms of family composition, the majority (85.6%) reported belonging to a heteroparental family, while 10.4% indicated a heteroparental family with separated/divorced parents. A smaller percentage identified as part of a homoparental family (0.4%), a homoparental family with separated/divorced parents (2.9%), or a single-parent family (0.7%). As for the highest education level of parent 1, the distribution was as follows: 1.9% with an elementary school diploma, 19.3% with a middle school diploma, 49.7% with a high school diploma, 5.4% with a bachelor’s degree, 11.2% with a master’s degree, 6.9% with a single-cycle master’s degree, and 5.6% with a postgraduate specialization. Concerning the highest education level of parent 2, 3.3% had an elementary school diploma, 30.2% a middle school diploma, 45.3% a high school diploma, 4.2% a bachelor’s degree, 6.2% a master’s degree, 6.9% a single-cycle master’s degree, and 3.9% a postgraduate specialization.

Data were collected during the period between March and April 2022 in ten secondary schools. The participation of each school was contingent upon the consent of the school head. Subsequently, the research was presented to students and parents, who received an information sheet with the study’s objectives, procedures, and consent norms. Students were informed of their voluntary participation, assuring them that non-participation would not incur any penalties. Following parental or guardian consent, interested students completed an online survey (developed using Qualtrics) during regular school hours. A researcher was present with the teacher during the entire survey administration, after previous collection of the informed consent form signed by students (if they were 18 years or older) or their parents (if they were minors). The time taken to complete the survey in each class was of maximum 30 min.

### Measures

#### Multidimensional State Boredom Scale (MSBS; [32, 67])

The Italian adolescent version by Spoto et al. [67] consists of 23 items measuring different aspects of state boredom (e.g., “Time is passing more slowly than usual,” “I am easily distracted,” “Everything seems to irritate me at the moment,” “Everything seems repetitive and routine to me,” “I wish time would go by faster”). Specifically, the items can be divided into five factors: perception of time (describing the slow passage of time), disengagement (i.e., lack of involvement), inattention (i.e., difficulty focusing on events), high arousal (and its negative effects), and internalizing aspects (associated with excessively low arousal). Participants are asked to respond on a 7-point Likert scale, ranging from 1 (“strongly disagree”) to 7 (“strongly agree”), indicating how they experience themselves and their lives at the precise moment they are responding, even if it involves feelings that differ from how they usually feel. The scores obtained for these five factors can be summed to obtain a total boredom score, which has been used in the present study. The Italian adolescent version of the MSBS showed an excellent reliability, with a McDonald’s ω coefficient for the total score of 0.90 [67].

#### Strength and Difficulties Questionnaire (SDQ; [29, 38])

It is a screening instrument used to assess psychological adjustment in children and adolescents. It consists of 25 items, divided into five subscales comprising five items each (e.g., “I often suffer from headache, stomachache or nausea,” “I have often anger crises or am in a bad mood,” “I am agitated, I can’t sit still for a long time,” “I am rather lonely, I tend to play alone,” “I try to be kind to others; I am respectful of their feelings”). Based on their experience over the past six months, participants are asked how true each statement is for them on a 3-point Likert scale ranging from 0 (“not true”) to 2 (“absolutely true”). Four subscales are designed to assess adjustment difficulties (i.e., emotional problems, conduct problems, hyperactivity-inattention, and problematic relationships with peers), while a subscale addresses prosocial behaviors. In the present study, we used two additional scales assessing internalizing and externalizing problems, which can be calculated by summing the scores obtained at the subscales of, respectively, emotional problems and problematic relationships with peers, and conduct problems and hyperactivity-inattention [29]. The Italian version of the SDQ presented a good reliability, with Cronbach’s alpha values ranging from 0.73 to 0.89 [29].

#### Difficulties in Emotion Regulation Scale (DERS; [35, 40])

It is a 36-item questionnaire to assess trait difficulties in emotion regulation. The items are divided into six scales or dimensions: non-acceptance of emotional responses (Nonacceptance), difficulties in engaging in goal-directed behavior (Goals), difficulties in impulse control (Impulse), lack of emotional awareness (Awareness), limited access to emotion regulation strategies (Strategies), and lack of emotional clarity (Clarity). Participants are asked to indicate how often each statement occurs to them on a 5-point Likert scale ranging from 1 (“almost never”) to 5 (“almost always”). The Italian version of the DERS showed good psychometric properties, with Cronbach’s alpha values ranging from 0.77 to 0.89 [35]. In the current research, only three out of the six scales (i.e., Goals, Impulse, and Strategies) were considered, for a total of 25 items (e.g., “When I am upset, I find difficulties at getting a job done,” “My emotions feel overwhelming and out of my control,” “I pay attention to the way I feel,” “When I am upset, I think I will stay in that state for a long time.”). We selected only these dimensions because they have been conceptualized as the most relevant and impulsive facets of emotion dysregulation, while the other ones can be considered as precursors of emotion dysregulation [19, 20]. Given that, in the present study, the Goals, Impulse, and Strategies scales were highly correlated (Goals-Impulse: *r* = .56, Goals-Strategies: *r* = .55, Impulse-Strategies: *r* = .68), they were combined into a single emotion dysregulation score.

#### Social Media Disorder Scale (SMDS; [75, 52])

This scale measures the level of problematic social media use by taking into account all the DSM-5 [5] criteria of addiction (i.e., concern about the object of addiction, development of tolerance, withdrawal symptoms, presence of relapse, altered mood, tendency to get into conflict with others when talking about social media, tendency to lie about time of use on social media, loss of interest in other hobbies or outside activities, and household-related conflicts). The scale used in this research is an abbreviated version of the SMDS, originally consisting of 27 items. This brief version consists of 9 items, each corresponding to a single criterion (e.g., “Did you find that you could think of nothing else but the time when you could use social media?”, “Did you often neglect other activities (e.g., hobbies and sports) because you wanted to use social media?”, “Did you often use social media to avoid negative emotions?”, and “Did you have serious conflicts with your parents and/or siblings because of your use of social media?”). Participants are asked to think about the past year and answer “yes” or “no” to each item. A total score of problematic social media use can be obtained by summing the score on each item. In the Italian version, the total score showed an acceptable reliability, with a Cronbach’s alpha of 0.72 [52].

### Data analysis

First, descriptive statistics (mean, standard deviations, skewness, and kurtosis) and correlation matrix of key variables were calculated. Furthermore, age and sex assigned at birth (henceforth sex, i.e., girl vs. boy) were tested as potential covariates by evaluating whether they were associated with the independent and dependent variables considered (i.e., DERS, MSBS, SMDS, SDQ internalizing and externalizing problems scales). Sex differences were based on the Mann-Whitney *U* test, as the assumptions of homogeneity of variance and normal distribution were violated, and the sample size of the two groups was markedly different.

**Fig. 1:**
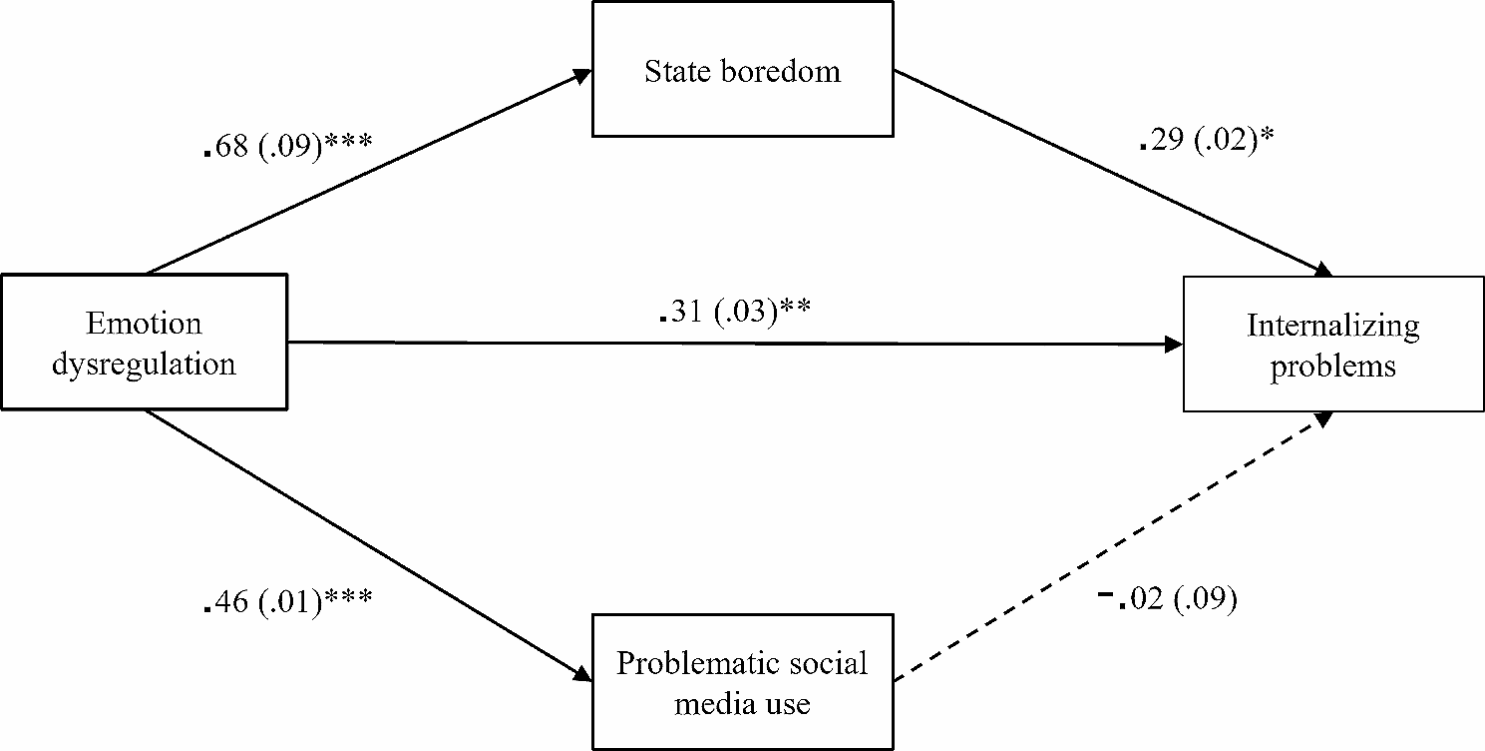
Standardized path coefficients of the model with the SDQ internalizing problems scale as the outcome variable. *Note*: Sex and age were added as covariates in the model; however, to simplify the representation, they were not included in the figure. Standard errors of the estimates are presented in brackets. * *p* < 0.05, ** *p* < 0.010, *** *p* < 0.001

**Fig. 2:**
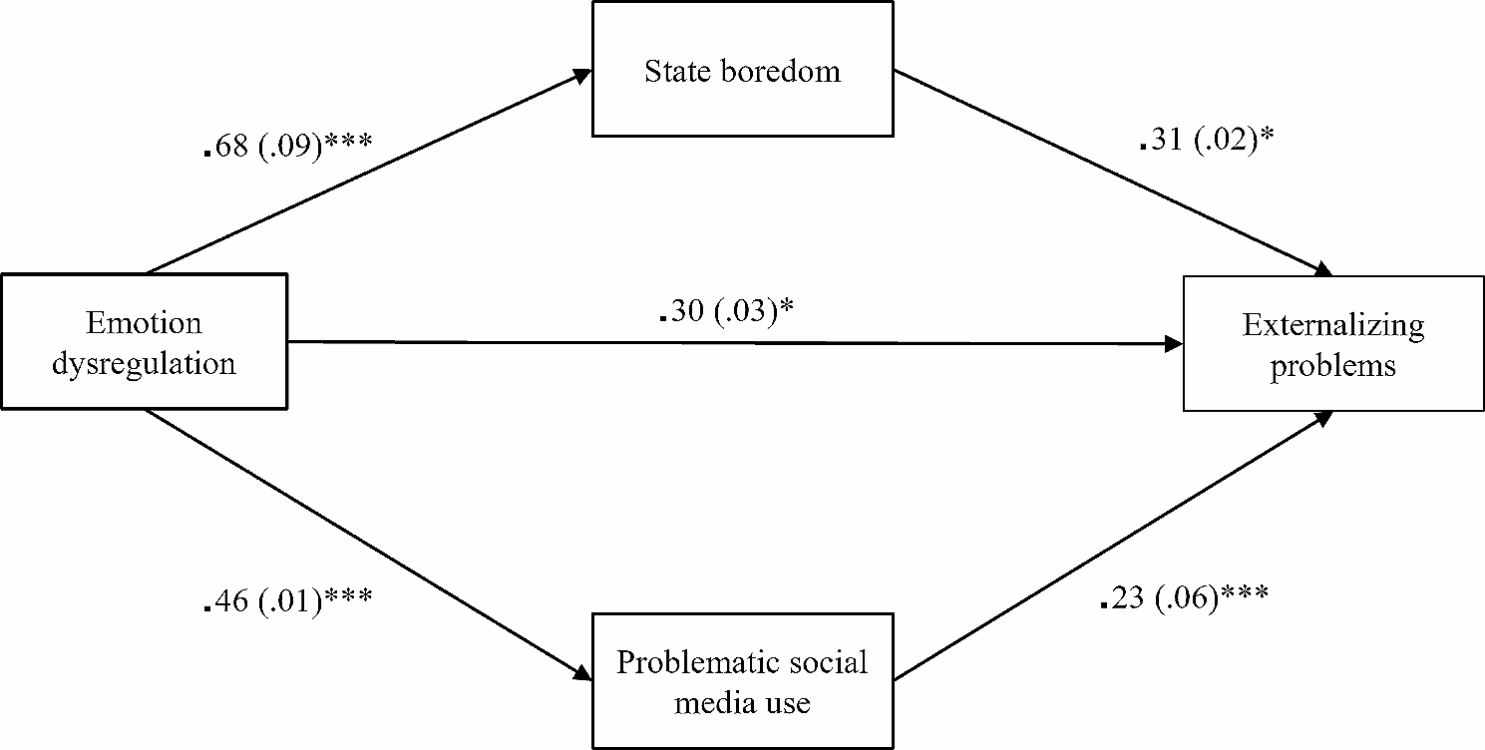
Standardized path coefficients of the model with the SDQ externalizing problems scale as the outcome variable. *Note*: Sex and age were added as covariates in the model; however, to simplify the representation, they were not included in the figure. Standard errors of the estimate are presented in brackets. * *p* < 0.05, *** *p* < 0.001

Then, two unnested path models were fitted to separately examine the effects of emotion dysregulation, state boredom, and problematic social media use on internalizing and externalizing problems. Specifically, the DERS score was inserted as the independent variable, while the MSBS and SMDS scores were input as mediators in both models. The two models instead differed for the outcome variable, which was the SDQ internalizing problems scale for the first model (Fig. 1) and the SDQ externalizing problems scale for the second model (Fig. 2). For indirect effects, small, medium, and large effect size thresholds were 0.02, 0.12, and.26, respectively [44].

Diagonally Weighted Least Squares (DWLS) was chosen as the estimator to account for nonnormality of the data. Model fit was evaluated considering the following fit indexes: Comparative Fit Index (CFI), Tucker-Lewis Index (TLI), Root-Mean-Square Error of Approximation (RMSEA), and Standardized Root-Mean-Square Residual (SRMR). CFI values close to 0.95 or greater, TLI values close to 0.90 or greater, RMSEA values close to 0.06 or below, and SRMR values close to 0.08 or below were considered indicative of a good model fit [47].

Path analyses were performed using the *lavaan* package [60] in R.

## Results

### Preliminary analyses

Descriptive statistics of the whole sample are reported in Table 1. Kurtosis and skewness values indicated that the data distribution had a slightly platykurtic and overall symmetrical shape. Moreover, all variables were significantly and positively interrelated, with moderate to large effects (Table 2). Pertaining to age and sex, the Mann-Whitney test pointed out significant sex differences on all the scales, with higher scores obtained by girls (Table 3). Instead, age resulted to be significantly correlated with the DERS and SMDS scores only (Table 2). On the basis of these results, age was inserted as a predictor of the DERS and SMDS, while sex was as a predictor of all the scales in the following path models.

**Table 1:** Descriptive statistics of the whole sample (*N* = 721)

	Mean (*SD*)	Kurtosis	Skewness
DERS	57 (16.5)	− 0.64	0.09
MSBS	99.1 (27.7)	− 0.13	− 0.37
SMDS	5.33 (2.57)	− 0.91	− 0.29
SDQ - IP	7.41 (3.92)	− 0.66	0.14
SDQ - EP	7.73 (3.46)	− 0.15	0.30

*Note*: DERS = Difficulties in Emotion Regulation Scale (score obtained by summing the single scores on the Strategies, Impulse, and Goals scales)

MSBS = Multidimensional State Boredom Scale, SMDS = Social Media Disorder Scale, SDQ = Strengths and Difficulties Questionnaire, IP = Internalizing Problems scale, EP = Externalizing Problems scale

**Table 2:** Pearson’s *r* correlations between the study variables (*N* = 721)

	1.	2.	3.	4.	5.	6.
1. DERS	1					
2. MSBS	0.69**	1				
3. SMDS	0.42**	0.43**	1			
4. SDQ - IP	0.60**	0.61**	0.28**	1		
5. SDQ - EP	0.58**	0.56**	0.44**	0.42**	1	
6. Age	0.09*	0.05	− 0.18**	0.05	− 0.04	1

*Note*: DERS = Difficulties in Emotion Regulation, MSBS = Multidimensional State Boredom Scale, SMDS = Social Media Disorder Scale, SDQ = Strengths and Difficulties Questionnaire, IP = Internalizing Problems scale, EP = Externalizing Problems scale

*p < 0.05, **p < 0.001

**Table 3:** Mean scores on the administered questionnaires for boys and girls, and results of the Mann-Whitney *U* test considering sex as the independent variable

	Girls (*n* = 466)			Boys (*n* = 250)			Sex differences		
	Mean	*SD*	*SE*	Mean	*SD*	*SE*	*U*	Rank biserial correlation	Comparison
DERS	61.6	15.9	0.74	49.3	14.9	0.94	34,499	0.41	F > M*
MSBS	106.8	25.1	1.17	84.4	26.4	1.67	30,995	0.47	F > M*
SMDS	5.71	2.43	0.11	4.62	2.7	0.17	44,477	0.24	F > M*
SDQ - IP	8.71	3.58	0.17	4.93	3.31	0.21	25,631	0.56	F > M*
SDQ - EP	8.05	3.46	0.16	7.1	3.39	0.21	48,919	0.16	F > M*

*Note*. DERS = Difficulties in Emotion Regulation Scale (score obtained by summing the single scores on the Strategies, Impulse, and Goals scales), MSBS = Multidimensional State Boredom Scale, SMDS = Social Media Disorder Scale, SDQ = Strengths and Difficulties Questionnaire, IP = Internalizing Problems scale, EP = Externalizing Problems scale

* p < 0.001

### Effects of emotion dysregulation, boredom, and problematic social media use on internalizing problems

The model examining the direct and indirect effects of the DERS on the SDQ internalizing problems scale, through the MSBS and SMDS, showed an excellent fit to the data (*χ*^2^ = 1480.58, *df* = 14, *p* < 0.001; CFI = 0.997, TLI = 0.987, RMSEA = 0.043, SRMR = 0.023). Overall, the model explained 46.9% of the variance for the SDQ internalizing problems scale and 11.9% for the DERS; explained variance for the mediators was 56.3% for the MSBS and 26.7% for the SMDS.

With regard to direct effects, the DERS and MSBS were significantly and positively linked to the SDQ internalizing problems scale, while no significant effect of the SMDS emerged (Fig. 1). Sex had a positive effect on the DERS (*β* = 0.34, *SE* = 1.25, *z* = 9.22, *p* < 0.001), MSBS (*β* = 0.16, *SE* = 3.03, *z* = 2.98, *p* = 0.003) and SDQ internalizing problems scale (*β* = 0.25, *SE* = 0.39, *z* = 5.19, *p* < 0.001), while age had a negative effect on the SMDS (*β* = − 0.21, *SE* = 0.06, *z* = -5.01, *p* < 0.001).

Finally, the indirect association of the DERS with the SDQ internalizing problems scale via the MSBS was significant and positive, showing a medium effect; on the contrary, no significant mediating effect of the SDMS was found (Table 4). The total effect emerged to be significant and positive as well, demonstrating a large effect (*β* = 0.50, *SE* = 0.01, *z* = 9.79, *p* < 0.001).

**Table 4:** Standardized indirect effects of emotion dysregulation on internalizing and externalizing problems via boredom and problematic social media use

Indirect paths	*β*	*SE*	*z*	*p*
DERS → MSBS → SDQ - IP	0.20	0.02	2.44	0.02
DERS → SMDS → SDQ - IP	− 0.01	0.01	− 0.36	0.72
DERS → MSBS → SDQ - EP	0.21	0.02	2.65	0.008
DERS → SMDS → SDQ - EP	0.11	0.01	4.34	< 0.001

*Note*: DERS = Difficulties in Emotion Regulation Scale, MSBS = Multidimensional State Boredom Scale, SMDS = Social Media Disorder Scale, SDQ = Strengths and Difficulties Questionnaire, IP = Internalizing Problems scale, EP = Externalizing Problems scale

### Effects of emotion dysregulation, boredom, and problematic social media use on externalizing problems

The model with the SDQ externalizing problems scale as the outcome variable was an excellent fit (*χ*^2^ = 1335.77, *df* = 14, *p* < 0.001; CFI = 0.997, TLI = 0.985, RMSEA = 0.045, SRMR = 0.024). Overall, the amount of variance explained by the model was 43.4% for the SDQ externalizing problems scale and 11.7% for the DERS; the mediators explained 56.3% of the variance for the MSBS and 27% for the SMDS.

The direct paths from the DERS, MSBS, and SMDS to the SDQ externalizing problems scale were all significant and positive (Fig. 2). Sex was a positive predictor of the DERS, MSBS, and SDQ externalizing problems scale scores (*β* = − 0.14, *SE* = 0.38, *z* = -2.72, *p* = 0.006), while age had a negative effect on the SMDS^1^.

Finally, both indirect relations were significant and positive, with a moderate effect for the MSBS and a small effect for the SMDS (Table 4). The total effect also resulted to be significant and positive with a large effect (*β* = 0.62, *SE* = 0.01, *z* = 11.02, *p* < 0.001).

## Discussion

Existing research widely recognizes the pivotal role of emotion dysregulation in the development of both internalizing and externalizing problems in adolescence [45, 54]. However, this body of evidence raises the question of whether there are different mechanisms through which emotion dysregulation is related to each type of problem. In this regard, two relevant factors are boredom and social media use, particularly considering that the level of boredom in adolescents and the importance of the Internet and social media for their lives have increased in recent years [52, 82]. Therefore, this study sought to address the above question by exploring the mediating role of state boredom and problematic social media use in the relation between emotion dysregulation and psychopathology, specifically differentiating between internalizing and externalizing problems.

Our results showed that emotion dysregulation was linked to internalizing and externalizing problems, both directly and indirectly. First, pertaining to direct relations, the positive associations observed would indicate that adolescents with elevated emotion dysregulation levels were also high in psychological problems levels. Specifically, given that the DERS measures trait emotion dysregulation, it could be that adolescents with dispositional deficits in adaptively regulating emotions are more prone to developing psychopathological symptoms of both the internalizing and externalizing spectra. This evidence is in line with the hypotheses and extant literature supporting the role of emotion dysregulation as a transdiagnostic vulnerability factor for psychopathology in general [45, 54].

Subsequently, in keeping with the hypotheses and prior research [11, 80, 81, 87], emotion dysregulation was a positive predictor of boredom and problematic social media use. As previously discussed, difficulties in emotion regulation may increase an adolescent’s susceptibility to boredom and engagement in dysfunctional social media use. Concerning the latter aspect, existing literature generally agrees on the fact that social media often serve as a means of emotion regulation in adolescence [18]. Specifically, following Goodman [39]’s theory, addictive behaviors can induce (temporary) pleasant emotions (i.e., positive reinforcement) while simultaneously alleviating distress (i.e., negative reinforcement); this mechanism seems to be also applicable to problematic social media use, which represents a potential addictive behavior [37, 85]. Therefore, adolescents who struggle to find an adaptive way to deal with emotional distress may turn to social media as a strategy to cope with and regulate their emotions. Additionally, the impulsivity trait may play a role in the link between emotion dysregulation and problematic social media use; indeed, impulsivity has been recognized as a predisposing or maintenance factor of dysfunctional technology-related behaviors [15, 58], and the inability to refrain from impulsive behavior when upset is a dimension of emotion dysregulation according to Gratz and Roemer [40]. The role of impulsivity may become relevant especially in adolescence, since phase-specific brain maturation processes seem to predispose adolescents to impulsivity and risk-taking behaviors [59]. Further studies are thereby recommended to delve deeper into this aspect.

As regards the link between emotion dysregulation and boredom, instead, the lack of research on this topic makes our result interesting and, at the same time, challenging to interpret thoroughly. A possible explanation builds upon and extends Bambrah et al. [11]’s work: adolescents facing challenges in controlling impulsive behaviors and acting in accordance with desired goals when experiencing negative emotions (which are facets of emotion dysregulation) may also have difficulties staying focused and engaged; this could foster boredom given that two of its main dimensions are inattention and lack of engagement [32]. Another consideration is that boredom has been conceptualized as a signal that a current activity or goal is not sufficiently rewarding, thus encouraging people to shift their focus to tasks with potentially more fruitful outcomes [12, 13]. Consequently, when confronted with unpleasant affective states, adolescents with difficulties regulating emotions and, more specifically, maintaining goal pursuit may perceive that goal as no longer meaningful; this could increase boredom levels and the individual’s need to engage in new activities. Moreover, boredom is inherently an unpleasant affective state [32, 78]; hence, adolescents who struggle to find and flexibly use appropriate strategies to regulate emotions may be unable to successfully deal with the distress associated with boredom, which can potentially intensify its manifestation. Finally, as outlined above for problematic social media use, impulsivity might be involved in the relation between emotion dysregulation and boredom; indeed, this factor has been considered a personality trait associated with boredom, as it negatively affects task engagement [27, 32, 55].

Notably, relevant differences emerged with regard to the indirect relations between emotion dysregulation and internalizing or externalizing problems, a result in part at odds with the hypotheses. In fact, while boredom partially mediated the associations between emotion dysregulation and both internalizing and externalizing problems, problematic social media use was a significant partial mediator only in the model with externalizing problems as the outcome. These findings highlight that the influence of emotion dysregulation on psychopathology can follow different paths and manifest in specific symptomatology depending on the interactions between various variables.

In particular, the multifaceted nature of boredom can contribute to making it a transdiagnostic factor. Boredom has both a low arousal dimension (e.g., “I feel down”) and a high arousal dimension (e.g., “I feel agitated”), which are more closely related to, respectively, internalizing and externalizing problems [67]. Therefore, as mentioned above, adolescents with emotion regulation difficulties may be more prone to experiencing boredom; this, in turn, could contribute to the development of psychopathology, which can take the form of internalizing or externalizing problems according to the predominant dimension of boredom in conjunction with other individual and environmental factors. Different response patterns to boredom may also affect whether young people develop internalizing or externalizing problems: for example, some adolescents may cope with boredom by attempting to control/suppress related emotional distress or isolating themselves, while others by engaging in risk-taking behaviors.

Problematic social media use, instead, would not seem to be a putative transdiagnostic factor, but rather a dimension-specific factor, as it emerged to be involved in the path from emotion dysregulation to externalizing problems only. This evidence extends beyond the results by Yang and Zhu [84], which showed that problematic Internet use played a significant role in fostering adolescent externalizing problems. The mediating role of problematic social media use in the relation between emotion dysregulation and externalizing problems could be understood by considering that using social media offers *immediate* gratification and relief from negative affective states, thus potentially representing a means of emotion regulation. In particular, adolescents with dispositional impulsivity, stimulation seeking, deficits in planning and inhibitory control, and preference for immediate reward over delayed gratification might be more inclined to use social media to modulate unpleasant emotions [70]; nevertheless, an excessive use of social media for emotion regulation purposes may contribute maintaining and/or worsening these traits, thus exposing young people to a higher risk of developing externalizing problems like conduct problems and hyperactivity-inattention symptoms [23, 46]. Furthermore, problematic social media use is currently conceptualized within the behavioral addiction framework [41, 52, 85]; thus, it could itself constitute an externalizing symptom that, if not properly recognized and treated, could evolve into a full-blown externalizing disorder.

Despite the interesting findings, the present study is not free from limitations. First, there was a clear sex imbalance in the sample, with a predominance of girls over boys. Then, the use of self-report questionnaires introduces the potential for biases related to social desirability, participants’ difficulty in accurately assessing themselves, and misinterpretation of questions; therefore, this topic should be explored further by including, for example, qualitative and/or other-report measures. Moreover, we did not use any measure of alexithymia, despite its possible involvement in the development of boredom [30, 51]; hence, future studies should include different measures of emotion dysregulation to provide a more nuanced perspective on this matter. Finally, the cross-sectional research design did not enable us to unravel the causal and temporal relations among the constructs. In particular, the results have been discussed in light of the dispositional nature of emotion dysregulation as measured by the DERS, thus conceiving emotion dysregulation as a possible precursor to boredom, problematic social media use, and internalizing and externalizing problems. Nevertheless, we cannot rule out that state boredom, problematic social media use, and pre-existing psychopathological symptoms hinder functional emotion regulation. The complex relations between these constructs may be a fruitful starting point for future research, preferably longitudinal. Importantly, subsequent studies should consider other variables (e.g., sensation seeking, negative affectivity, self-control) that could affect the associations between emotion dysregulation, boredom, social media use, and psychopathology [14]. Lastly, to corroborate our findings about the role of boredom as a transdiagnostic vulnerability factor, future investigations should examine this topic by including trait boredom as well; indeed, this would provide a more comprehensive understanding of boredom’s potential influence across different psychopathological dimensions.

### Conclusions and practical implications

This study’s exploration of a hitherto overlooked but fundamental topic can provide important insights into the understanding of adolescent psychopathology, also offering valuable implications for clinical practice.

First, emotion dysregulation was confirmed to be associated with both internalizing and externalizing problems, as well as with state boredom and problematic social media use. Clinically, this result underscores the importance of assessing and targeting emotion dysregulation in transdiagnostic interventions aimed at preventing psychopathology and promoting psychological well-being in young people.

Then, boredom emerged as a significant factor associated with internalizing and externalizing problems, also acting as a partial mediator in the relation between emotion dysregulation and both psychopathological dimensions. This suggests a transdiagnostic nature of boredom, emphasizing its relevance beyond a phase-specific affective state; indeed, it can act as a risk factor for the development/exacerbation of psychopathology and/or a symptom of severe underlying distress [67]. Consequently, mental health treatment and preventive programs in adolescence should incorporate modules that assess and target boredom; more specifically, strategies to equip adolescents with functional strategies to cope with boredom should be emphasized, especially in cases of comorbid internalizing and externalizing symptoms.

Finally, the fact that emotion dysregulation was found to increase externalizing problems levels through problematic social media use speaks to the importance of responsible Internet use in adolescence. In this regard, school-based preventive efforts on digital media use (e.g., media literacy programs) can play a crucial role in educating adolescents about healthier social media usage, thus reducing mental health risks associated with problematic online behaviors [79]. In addition, interventions for adolescents with externalizing problems should extend beyond symptom reduction to investigate patterns of Internet and social media use, as a maladaptive use could contribute to maintaining psychopathology and reducing treatment effectiveness over time.

## Data Availability

The data that support the findings of this study are available from the corresponding author, S.I., upon reasonable request.

## Footnotes

1 The estimates of the effect of sex on the DERS and MSBS and of age on the SMDS were the same as outlined in the previous model.

## References

[1] Achenbach TM (1991). Manual for the child Behavior Checklist 04–18 and 1991 profile.

[2] Achenbach TM, Ivanova MY, Rescorla LA, Turner LV, Althoff RR (2016). Internalizing/externalizing problems: review and recommendations for clinical and research applications. J Am Ac Child Adol Psych.

[3] Alda M, Minguez J, Montero-Marin J, Gili M, Puebla-Guedea M, Herrera-Mercadal P, Navarro-Gil M, Garcia-Campayo J (2015). Validation of the Spanish version of the Multidimensional State Boredom Scale (MSBS). Health Qual Life Outcomes.

[4] Aldao A, Nolen-Hoeksema S, Schweizer S (2010). Emotion-regulation strategies across psychopathology: a meta-analytic review. Clin Psychol Rev.

[5] American Psychiatric Association (2013). Diagnostic and statistical manual of mental disorders.

[6] Andersen SL, Teicher MH (2008). Stress, sensitive periods and maturational events in adolescent depression. Trends Neurosc.

[7] Andreassen CS, Billieux J, Griffiths MD, Kuss DJ, Demetrovics Z, Mazzoni E, Pallesen S (2016). The relationship between addictive use of social media and video games and symptoms of psychiatric disorders: a large-scale cross-sectional study. Psychol Addict Behav.

[8] Andreassen CS, Pallesen S (2014). Social network site addiction–An overview. Curr Pharm Des.

[9] Andreassen CS, Pallesen S, Griffiths MD (2017). The relationship between addictive use of social media, narcissism, and self-esteem: findings from a large national survey. Addict Behav.

[10] Atherton OE, Lawson KM, Robins RW (2020). The development of effortful control from late childhood to young adulthood. J Pers Soc Psychol.

[11] Bambrah V, Wyman A, Eastwood JD (2023). A longitudinal approach to understanding boredom during pandemics: the predictive roles of trauma and emotion dysregulation. Front Psychol.

[12] Bench SW, Lench HC (2013). On the function of boredom. Behav Sci.

[13] Bench SW, Lench HC (2019). Boredom as a seeking state: Boredom prompts the pursuit of novel (even negative) experiences. Emotion.

[14] Bieleke M, Wolff W, Keller L (2022). Getting trapped in a dead end? Trait self-control and boredom are linked to goal adjustment. Motivation and Emotion.

[15] Billieux J, Van der Linden M, Rochat L (2008). The role of impulsivity in actual and problematic use of the mobile phone. App Cogn Psychol.

[16] Biolcati R, Mancini G, Trombini E (2018). Proneness to boredom and risk behaviors during adolescents’ free time. Psychol Rep.

[17] Blakemore S-J (2019). Adolescence and mental health. Lancet.

[18] Blumberg FC, Rice JL, Dickmeis A. Social media as a venue for emotion regulation among adolescents. In: Tettegah SJ, editor. Emotions, technology, and social media. Elsevier Ac Press; 2016. pp. 105–16.

[19] Bornovalova MA, Ouimette P, Crawford AV, Levy R (2009). Testing gender effects on the mechanisms explaining the association between post-traumatic stress symptoms and substance use frequency. Addict Behav.

[20] Bottesi G, Ghisi M, Caggiu I, Lauriola M (2021). How is intolerance of uncertainty related to negative affect in individuals with substance use disorders? The role of the inability to control behaviors when experiencing emotional distress. Addict Behav.

[21] Buhrmester D (1990). Intimacy of friendship, interpersonal competence, and adjustment during preadolescence and adolescence. Child Dev.

[22] Chin A, Markey A, Bhargava S, Kassam KS, Loewenstein G, Washington (2017). D C).

[23] Chou WJ, Liu TL, Yang P, Yen CF, Hu HF (2015). Multi-dimensional correlates of internet addiction symptoms in adolescents with attention-deficit/hyperactivity disorder. Psych Res.

[24] Cole PM, Hall SE, Beauchaine TP, Hinshaw SP (2008). Emotion dysregulation as a risk factor for psychopathology. Child and adolescent psychopathology.

[25] Cracco E, Goossens L, Braet C (2017). Emotion regulation across childhood and adolescence: evidence for a maladaptive shift in adolescence. Eur Child Adol Psych.

[26] Crone EA, Dahl RE (2012). Understanding adolescence as a period of social-affective engagement and goal flexibility. Nat Rev Neurosci.

[27] Dahlen ER, Martin RC, Ragan K, Kuhlman MM (2004). Boredom proneness in anger and aggression: effects of impulsiveness and sensation seeking. Pers Indiv Diff.

[28] Dickstein DP, Leibenluft E (2006). Emotion regulation in children and adolescents: boundaries between normalcy and bipolar disorder. Dev Psychopathol.

[29] Di Riso D, Salcuni S, Chessa D, Raudino A, Lis A, Altoè G (2010). The strengths and difficulties Questionnaire (SDQ). Early evidence of its reliability and validity in a community sample of Italian children. Pers Indiv Diff.

[30] Eastwood JD, Cavaliere C, Fahlman SA, Eastwood AE (2007). A desire for desires: Boredom and its relation to alexithymia. Pers Indiv Diff.

[31] Erikson E (1968). Identity, youth, and crisis.

[32] Fahlman SA, Mercer-Lynn KB, Flora DB, Eastwood JD (2013). Development and validation of the multidimensional state boredom scale. Assessment.

[33] Freund VA, Schulenberg JE, Maslowsky J (2021). Boredom by sensation-seeking interactions during adolescence: associations with substance use, externalizing behavior, and internalizing symptoms in a US national sample. Prev Sci.

[34] Gatta M, Angelico C, Rigoni F, Raffagnato A, Miscioscia M (2022). Alexithymia and psychopathological manifestations centered on the body: somatization and self-harm. J Clin Med.

[35] Giromini L, Velotti P, de Campora G, Bonalume L, Cesare Zavattini G (2012). Cultural adaptation of the difficulties in emotion regulation scale: reliability and validity of an Italian version. J Clin Psychol.

[36] Gioia F, Rega V, Boursier V (2021). Problematic internet use and emotional dysregulation among young people: a literature review. Clin Neuropsych.

[37] Giordano AL, Schmit MK, McCall J (2023). Exploring adolescent social media and internet gaming addiction: the role of emotion regulation. J Addict off Couns.

[38] Goodman R (1997). The strengths and difficulties Questionnaire: a research note. J Child Psychol Psych all Disc.

[39] Goodman A (2001). What’s in a name? Terminology for designating a syndrome of driven sexual behavior. Sex Add Compuls.

[40] Gratz KL, Roemer L (2004). Multidimensional assessment of emotion regulation and dysregulation: development, factor structure, and initial validation of the difficulties in emotion regulation scale. J Psychopathol Beh Assess.

[41] Griffiths MD (2013). Social networking addiction: emerging themes and issues. J Add Res Ther.

[42] Griffiths MD, Kuss DJ, Billieux J, Pontes HM (2016). The evolution of internet addiction: a global perspective. Addict Behav.

[43] Gullone E, Hughes EK, King NJ, Tonge B (2010). The normative development of emotion regulation strategy use in children and adolescents: a 2-year follow-up study. J Child Psychol Psychiatry Allied Discip.

[44] Hayes AF, Rockwood NJ (2017). Regression-based statistical mediation and moderation analysis in clinical research: observations, recommendations, and implementation. Beh Res Ther.

[45] Helland SS, Mellblom AV, Kjøbli J, Wentzel-Larsen T, Espenes K, Engell T, Kirkøen B (2022). Elements in mental health interventions associated with effects on emotion regulation in adolescents: a meta-analysis. Admin Pol Mental Health.

[46] Herba CM, Tranah T, Rubia K, Yule W (2006). Conduct problems in adolescence: three domains of inhibition and effect of gender. Dev Neuropsych.

[47] Hu L-T, Bentler PM (1999). Cutoff criteria for fit indexes in covariance structure analysis: conventional criteria versus new alternatives. Struct Eq Mod.

[48] Kietzmann JH, Hermkens K, McCarthy IP, Silvestre B (2011). Social media? Get serious! Understanding the functional building blocks of social media. Bus Horiz.

[49] Ivie EJ, Pettitt A, Moses LJ, Allen NB (2020). A meta-analysis of the association between adolescent social media use and depressive symptoms. J Aff Dis.

[50] Lewinsohn PM, Striegel-Moore RH, Seeley JR (2000). Epidemiology and natural course of eating disorders in young women from adolescence to young adulthood. J Am Ac Child Adol Psych.

[51] Liu Y, Chen L, Wang Z, Guo G, Zhang M, Chen S (2022). Role of alexithymia in predicting internet novel addiction through boredom proneness. Int J Env Res Pub Health.

[52] Marino C, Gini G, Angelini F, Vieno A, Spada MM (2020). Social norms and e-motions in problematic social media use among adolescents. Add Beh Rep.

[53] Marino C, Lenzi M, Canale N, Pierannunzio D, Dalmasso P, Borraccino A, Cappello N, Lemma P, Vieno A, 2018 HBSC-Italia Group, the 2018 HBSC-Italia Group (2020). Problematic social media use: associations with health complaints among adolescents. Ann Ist Sup Sanità.

[54] McLaughlin KA, Hatzenbuehler ML, Mennin DS, Nolen-Hoeksema S (2011). Emotion dysregulation and adolescent psychopathology: a prospective study. Beh Res Ther.

[55] Mercer-Lynn KB, Flora DB, Fahlman SA, Eastwood JD (2013). The measurement of boredom: differences between existing self-report scales. Assessment.

[56] Nikkelen SW, Valkenburg PM, Huizinga M, Bushman BJ (2014). Media use and ADHD-related behaviors in children and adolescents: a meta-analysis. Dev Psychol.

[57] Ochsner KN, Gross JJ (2008). Cognitive emotion regulation: insights from social cognitive and affective neuroscience. Curr Dir Psychol Sci.

[58] Pérez de Albéniz Garrote G, Rubio L, Medina Gómez B, Buedo-Guirado C (2021). Smartphone abuse amongst adolescents: the role of impulsivity and sensation seeking. Front Psychol.

[59] Romer D (2010). Adolescent risk taking, impulsivity, and brain development: implications for prevention. Dev Psychobiol.

[60] Rosseel Y (2012). Lavaan: an R Package for Structural equation modeling. J Stat Soft [Internet].

[61] Schwartze MM, Frenzel AC, Goetz T, Pekrun R, Reck C, Marx AKG, Fiedler D (2021). Boredom makes me sick: adolescents’ boredom trajectories and their health-related quality of life. Int J Env Res Pub Health.

[62] Shannon H, Bush K, Villeneuve PJ, Hellemans KG, Guimond S (2022). Problematic social media use in adolescents and young adults: systematic review and meta-analysis. JMIR Ment Health.

[63] Sherman LE, Michikyan M, Greenfield PM (2013). The effects of text, audio, video, and in-person communication on bonding between friends. Cyberpsychol J Psychosoc Res Cyberspace.

[64] Somerville LH, Jones RM, Casey BJ (2010). A time of change: behavioral and neural correlates of adolescent sensitivity to appetitive and aversive environmental cues. Brain Cogn.

[65] Spaeth M, Weichold K, Silbereisen RK (2015). The development of leisure boredom in early adolescence: predictors and longitudinal associations with delinquency and depression. Dev Psychol.

[66] Spear LP (2009). Heightened stress responsivity and emotional reactivity during pubertal maturation: implications for psychopathology. Dev Psychopathol.

[67] Spoto A, Iannattone S, Valentini P, Raffagnato A, Miscioscia M, Gatta M (2021). Boredom in adolescence: validation of the Italian version of the Multidimensional State Boredom Scale (MSBS) in adolescents. Children.

[68] Steinberg L, Morris AS (2001). Adolescent development. Annu Rev Psychol.

[69] Tam KYY, Chan CS, van Tilburg WAP, Lavi I, Lau JYF (2022). Boredom belief moderates the mental health impact of boredom among young people: Correlational and multi-wave longitudinal evidence gathered during the COVID-19 pandemic. J Pers.

[70] Taylor S, Pattara-Angkoon S, Sirirat S, Woods D. The theoretical underpinnings of internet addiction and its association with psychopathology in adolescence. Int J Adol Med Health. 2017;31(5). 10.1515/ijamh-2017-0046/j/ijamh.2019.31.issue-5/ijamh-2017-0046/ijamh-2017-0046.xml. 10.1515/ijamh-2017-0046 28682784

[71] Thompson RA (2019). Emotion dysregulation: a theme in search of definition. Dev Psychopathol.

[72] Tsitsika A, Critselis E, Louizou A, Janikian M, Freskou A, Marangou E, Kormas G, Kafetzis DA (2011). Determinants of internet addiction among adolescents: a case-control study. ScientificWorld.

[73] Valkenburg PM, Peter J (2008). Adolescents’ identity experiments on the internet: consequences for social competence and self-concept unity. Commun Res.

[74] Valkenburg PM, Peter J (2011). Online communication among adolescents: an integrated model of its attraction, opportunities, and risks. J Adolesc Health.

[75] van den Eijnden RJJM, Lemmens JS, Valkenburg PM (2016). The Social Media Disorder Scale. Comp Hum Beh.

[76] Van Lier PAC, Vitaro F, Barker ED, Brendgen M, Tremblay RE, Boivin M (2012). Peer victimization, poor academic achievement, and the link between childhood externalizing and internalizing problems. Child Dev.

[77] van Rooij AJ, Schoenmakers TM, van de Eijnden RJ, van de Mheen D (2010). Compulsive internet use: the role of online gaming and other internet applications. J Adolesc Health.

[78] van Tilburg WAP, Igou ER (2012). On boredom: lack of challenge and meaning as distinct boredom experiences. Motiv Em.

[79] Walther B, Hanewinkel R, Morgenstern M (2014). Effects of a brief school-based media literacy intervention on digital media use in adolescents: Cluster randomized controlled trial. Cyberpsychol Beh Soc Network.

[80] Wartberg L, Thomasius R, Paschke K (2021). The relevance of emotion regulation, procrastination, and perceived stress for problematic social media use in a representative sample of children and adolescents. Comp Hum Beh.

[81] Weybright EH, Doering EL, Perone S (2022). Difficulties with emotion regulation during COVID-19 and associations with boredom in college students. Beh Sci.

[82] Weybright EH, Schulenberg J, Caldwell LL (2020). More bored today than yesterday? National trends in adolescent boredom from 2008 to 2017. J Adol Health.

[83] Woods HC, Scott H, #Sleepyteens (2016). Social media use in adolescence is associated with poor sleep quality, anxiety, depression and low self-esteem. J Adol.

[84] Yang S, Zhu X (2023). How does problematic internet use influence Chinese rural adolescent externalizing problem behaviors? The mediating role of mental health and the moderating role of parental knowledge. Int J Env Res Pub Health.

[85] Young K, Montag C, Reuter M (2015). The evolution of internet addiction disorder. Internet addiction, studies in neuroscience, psychology, and behavioral economics.

[86] Zimmermann P, Iwanski A (2014). Emotion regulation from early adolescence to emerging adulthood and middle adulthood. Int J Beh Dev.

[87] Zsido A, Arató N, Láng A, Labadi B, Pakai-Stecina D, Bandi S (2021). The role of maladaptive cognitive emotion regulation strategies and social anxiety in problematic smartphone and social media use. Pers Indiv Diff.

